# The effect of COVID-19 on prostate cancer testing in Australia

**DOI:** 10.1186/s12894-022-01043-2

**Published:** 2022-06-21

**Authors:** Anika Jain, James Macneil, Lawrence Kim, Manish I. Patel

**Affiliations:** 1 Department of Urology, Westmead Hospital, Western Sydney Local District, PO Box 533, Wentworthville, NSW 2145 Australia; 2 Faculty of Medicine, Macquarie University, Sydney, Australia; 3 Faculty of Medicine, The University of Sydney, Sydney, Australia

**Keywords:** PSA testing, COVID 19 trends, Australia

## Abstract

**Aim:**

The effects of the COVID-19 pandemic on healthcare in Australia have yet to be fully determined. There are well documented decreases in the rates of screening and diagnostic testing for many cancers in 2020, with commensurate stage migration of cancers when they are eventually detected. We aimed to determine whether there was a decrease in the rate of prostate cancer (PC) screening and testing in Australia in 2020.

**Method:**

Data was extracted from the Department of Human Services (DHS) website for Medicare Benefits Schedule (MBS) item numbers for tests pertinent to detection of Prostate Cancer. This data is de-identified and publicly available. Data was analysed at both a national, and a state level.

**Results:**

For 2020 nationwide the percentage change for prostate cancer testing was minor with 97% as many PSA tests, 99% as many prostate MRIs, and 105% as many prostate biopsies as the average for the preceding years. The differences were not significant (PSA tests *p* = 0.059 and prostate biopsies *p* = 0.109). The predicted values are fairly similar to both the average values for the preceding 5 years and the actual number of tests done in 2020. With exception of PSA tests in Victoria the actual number of tests performed was within the 95% Prediction Interval (performed: 167,426; predicted 171,194–196,699; *p* = 0.015).

**Conclusion:**

The current pandemic has had a widespread reach across Australia, with varying impact across each state and territory. Contrary to the trends across the world, our data suggest that during 2020 in Australia most areas remained unaffected in terms of prostate cancer testing excluding Victoria, which had statistically significant decrease in the number of PSA tests correlating with the extended lockdown that occurred in the state.

## Aim

The effects of the COVID-19 pandemic on healthcare in Australia have yet to be fully determined. The collateral damage of the pandemic is likely to have a severe impact on healthcare [1]. A confluence of factors are contributing to impair access to cancer detection and treatment services: lockdowns, patient reluctance to access healthcare, and the direct effect of the COVID-19 pandemic on healthcare systems. This has resulted in a well-documented decrease in the rates of screening and diagnostic testing for many cancers in 2020 in Australia and overseas, with commensurate stage migration of cancers when they are eventually detected [2, 3].

Early evidence from overseas has suggested decreased levels of prostate specific antigen (PSA) testing in highly impacted communities [4], and decreased diagnosis of various urological cancers including prostate cancer [5]. Anecdotally many Australian urologists fear that there are men with potentially curable prostate cancers that will have missed their opportunity for cure because of decreased uptake of screening tests during the lockdowns of 2020.

Although the true impact of COVID-19 on Prostate Cancer in Australia will not be known for many years owing to the natural history of the disease, it is possible using Medicare Benefits Schedule (MBS) to estimate the rate of prostate cancer (PC) screening and testing in Australia in 2020. We aimed to determine whether or not the testing rate in Australia had differed significantly from the pre-pandemic rates. We also aimed to demonstrate the effect of the regional lockdowns which have characterized the Australian response to the COVID-19 pandemic.

## Method

Data was extracted from the Department of Human Services (DHS) website for Medicare Benefits Schedule (MBS) item numbers for tests pertinent to detection of Prostate Cancer. MBS item numbers are collected on all eligible procedure or tests performed on eligible Australian citizens and residents regardless of insurance status (i.e. it is collected on patient ion both public and private settings, well over 95% of the Australian population is covered by this system). This data is deidentified, and is publicly available (available at: http://medicarestatistics.humanservices.gov.au/statistics/mbs_item.jsp).

These item numbers were the 66,655 (defined as “prostate specific antigen quantification, one item reimbursed in a 12-month period”, referred to hereafter as “PSA test”), 63,541(defined as “Multiparametric Magnetic Resonance Imaging scan of the prostate for the detection of cancer”, referred to hereafter as “prostate MRI”). We also looked at item number 37219 (defined as “Prostate or prostatic bed, needle biopsy of, by the transperineal route”, referred to here after as “prostate biopsy”), which could be used either in the initial detection of prostate cancer or in surveillance of men with existing diagnoses. Quantities of each of these tests performed in each state and territory were extracted from the DHS website per calendar year (i.e. 1 January to 31 December), and per month between January 2015 and December 2020 (expect for prostate MRI for which data only exists after July 2018). Data was extracted 4 July 2021.

In order to predict the test usage for 2020, linear regressions were performed on the rate of these diagnostic tests against the population as reported by the Australian Bureau of Statistics. (https://www.abs.gov.au/statistics/people/population/national-state-and-territory-population).

Data was analysed at both a national, and a state level. All analysis was performed using PSPP version 1.4.0 (GNU Project (2020). Free Software Foundation. Boston, MA).

## Results

Over the six-years of data analysed a total of 4,048,099 PSA tests, and 118,328 prostate biopsies were performed. Over the period 2019–2020 there were 68,429 prostate MRIs. For the years 2015–2019 on average 678,082 PSA tests and 19,573 prostate biopsies were performed on average per annum.

**Fig. 1:**
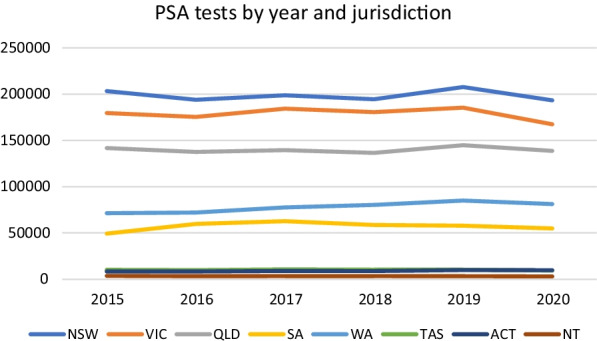
PSA tests by year and jurisdiction

**Fig. 2:**
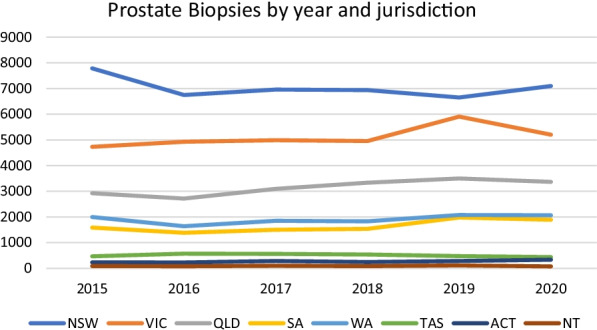
Prostate biopsies by year and jurisdiction

As shown in Table 1, for 2020 nationwide the percentage change for each of these was minor with 97% as many PSA tests, 99% as many prostate MRIs, and 105% as many prostate biopsies as the average for the preceding years. The differences were not statistically significant. As shown in Figs. 1 and 2, the number of PSA tests and prostate biopsies are relatively consistent over the period 2015–2020 within each jurisdiction.

**Table 1:** Average prostate cancer detection tests nationally 2015–2019 and tests in 2020

	Average 2015–2019 (95%CI)	2020	Difference (95%CI; and % of average)	Significance
PSA tests	678,082 (660,114 to 704,283)	657,687	− 20,395 (− 41,991 to 1200; 97)	*P* = 0.059 (t = 2.62)
Prostate MRI	34,368^a^	34,061	− 307 (†; 99)	N/A†
Prostate biopsies	19,573 (18,285 to 20,980)	20,463	+ 890 (314–2094; 105)	*P* = 0.109 (t = − 2.05)

^†^Statistical analysis not possible owing to limited data set

^a^Full year data only available for 2019–2020

A sub- group analysis was conducted for the use of PSA testing and prostate biopsies in Victoria, shown in Table 2. There was a statistically significant decrease in the number of PSA tests conducted in Victoria in 2020 as compared to average for the preceding 5 years (167,426 compared to 181,056), and a non-significant increase in the number of prostate biopsies (5201 compared to 5101). Fewer prostate MRIs were performed (8683 compared to 9826), however it is not possible to say if the difference was significant.

**Table 2:** Average prostate cancer detection tests in Victoria 2015–2019 and tests in 2020

	Average 2015–2019 (95%CI)	2020	Difference (95%CI; and % of average)	Significance
PSA tests	181,056 (175,425 to 185,379)	167,426	− 13,629 (8671–18,588; 92)	*P* = 0.002 (t = 7.63)
Prostate MRI*	9826†	8683	− 1143 (‡; 88)	N/A‡
Prostate biopsies	5101 (4730–5906)	5201	+ 100 (− 472 to 672; 102)	*P* = 0.653 (t = − 0.49)

^*^Data from DHS grouped for Victoria and Tasmania

^†^Full year data only available for 2019–2020

^‡^Statistical analysis not possible owing to limited data set

**Fig. 3:**
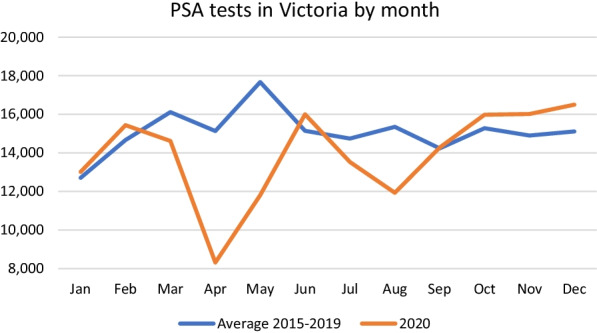
PSA tests in Victoria by month

A further analysis was conducted by month for PSA tests which showed statistically significant decreases in the number of tests performed in March to May and again August, which correlate to the periods of maximal restrictions in the first and second Victorian lockdowns (respectively 16 March to 11 May and 20 June to 26 October). There were statistically significant increases in the number of PSA tests in November. The analysis and trends are shown in Table 3 and Fig. 3 respectively.

**Table 3:** PSA testing in Victoria by month

	Average 2015–2019 (95%CI)	2020	Difference (95%CI; and % of average)	Significance
January	12,706 (9890–16,852)	13,015	+ 309 (− 3004–3622; 102)	*P* = 0.809 (t = − 0.26)
February	14,666 (12,062–16,136)	15,439	+ 773 (− 1264 to 2811; 105)	*P* = 0.352 (t = − 1.05)
March	16,114 (14,954–17,254)	14,618	− 1496 (473 to − 2518; 91)	*P* = 0.015 (t = 4.06)
April	15,133 (14,073–15,909)	8313	− 6820 (− 5854 to − 7785; 55)	*P* < 0.001 (t = 19.61)
May	17,674 (15,427–20,242)	11,803	− 5871 (− 3686 to − 8055; 67)	*P* = 0.002 (t = 7.46)
June	15,145 (13,953–16,274)	16,003	+ 858 ( − 292 to 2007; 105)	*P* = 0.107 (t = − 2.07)
July	14,742 (13,124–15,930)	13,528	− 1214 (− 91 to − 2519; 91)	*P* = 0.061 (t = 2.58)
August	15,350 (14,347–16,072)	11,932	− 3418 (− 4262 to − 2572; 77)	*P* < 0.001 (t = 11.23)
September	14,233 (13,048–15,118)	14,278	+ 45 (− 920 to 1009; 100)	*P* = 0.904 (t = − 0.13)
October	15,281 (12,650–16,947)	15,981	+ 700 (− 1285 to 2685;105)	*P* = 0.383 (t = − 0.98)
November	14,902 (14,171–15,302)	16,015	+ 1113 (512 –1714; 107)	*P* = 0.007 (t = − 5.14)
December	15,110 (13,312–16,799)	16,501	+ 1391 (226–3009; 109)	*P* = 0.75 (t = − 2.39)

A further analysis was conducted to estimate the number of PSA tests and biopsies which could be expected in 2020 based on population. These results are shown in Table 4 (national data) and Table 5 (Victorian data).

**Table 4:** predicted prostate cancer detection tests performed nationally based on linear regression of tests performed in 2015–2019 compared to tests performed in 2020

	Predicted tests in 2020 (95% Prediction Interval)	R^2^ of model	Tests performed in 2020	Difference (% of predicted)	Significance
PSA tests	698,036 (568,163–827,909)	0.59	657,687	− 40,349 (94)	*P* = 0.379 (t = 0.99)
Prostate biopsies	20,353 (10,811–29,896)	0.29	20,463	+ 110 (100)	*P* = 0.973 (t = − 0.036)

**Table 5:** Predicted prostate cancer detection tests performed in Victoria based on linear regression of tests performed in 2015–2019 compared to tests performed in 2020

	Predicted tests in 2020 (95% prediction interval)	R^2^ of model	Tests performed in 2020	Difference (% of predicted)	Significance
PSA tests	183,946 (171,194–196,699)	0.37	167,426	− 16,520 (91)	*P* = 0.015 (t = 4.12)
Prostate biopsies	5522 (4338–6706)	0.60	5201	− 321 (94)	*P* = 0.437 (t = 0.86)

As can be seen the predicted values are fairly similar to both the average vales for the preceding 5 years and the actual number of tests done in 2020. With exception of PSA tests in Victoria the actual number of tests performed was within the 95% Prediction Interval. This was also the only case in which the actual number of tests performed was significantly different from the prediction (*P* = 0.015).

## Discussion

While there are studies under way to characterize the direct effects of the COVID-19 pandemic on the care of patients with cancer [1–3], there have been few quantitative reports of the impact the pandemic has had on the normal course of prostate cancer diagnosis and treatment encounters in Australia. Outside of Australia data from highly impacted communities suggests a significant impact on the rate and pattern of prostate cancer diagnosis [6]. In Italy, De Vincentiis et al. reported that the number of prostate cancer diagnoses was decreased by − 75% in 2020 compared to the average number recorded in the previous two years at a secondary care hospital network in central Italy [5]. Another study from Northern Italy, Verona had congruent findings reporting a − 62% decrease in PSA testing as compared to previous years [4]. Similarly, a retrospective study analysing the US population by Chen et al. reported decline in prostate cancer testing by − 79.3% as compared to the previous year from March through to May 2020 [7].

The picture in Australia has so far not been well described. Cancer Australia published a 6-month period of data for prostate cancer testing from January to June 2020 compared to the data from the same period in 2019 [8] which suggested a significant decrease in the rate of testing. However, our data includes a more in-depth analysis over the whole of 2020, and makes a comparison with the data from 2015 to 2019. The trends suggested in the Cancer Australia report do not appear to have continued in the second half of 2020.

Despite the limitations of our study, we do feel that the results show a marked difference in the Australian context. For the whole year of 2020, for PSA tests and biopsies the numbers performed were within 10% of the average performed in the preceding 5 years and the predicted test usage based on modelling (a larger difference was shown in MRI tests in Victoria, however as this is a comparison with 2019 alone, the significance of this cannot be assumed). With the exception of PSA testing in Victoria the number of tests performed was within the 95% Prediction Interval, and this was also the only test for which a statistically significant drop could be shown as compared to the average or the prediction.

Interestingly, by looking at a month by month analysis we can see that the rate of PSA testing in Victoria does correlate quite closely with the periods of peak first and second lockdown in Melbourne, (respectively 16 March to 11 May and 20 June to 26 October) though from the available data being more affected in the first rather than the second lockdowns. We cannot explain with the available data why this discrepancy between the lockdowns exists. Whether or not the same pattern will be demonstrated after the Sydney and Melbourne lockdowns for the 2021 Delta-outbreak remains to be seen.

It is interesting to speculate why the picture in Australia is so much different from the rest of the world. Although there is no doubt that there was less impact on the health system from the COVID-19 pandemic [9], Australian jurisdictions implement some of the harshest lockdowns anywhere in the world [10]. Our current study does not allow us to tease out the reasons for this, however given the manifest risk of severely impacting testing for prostate cancer we believe it should be treated as a successful outcome for the Australian healthcare system.

As a result of the impact of the COVID-19 pandemic on rate of prostate cancer detection in the rest of the world, changes are occurring in the guidelines for prostate cancer testing and treatment [11–14]. These all recommend taking a risk adjusted approach to managing prostate cancer in light of resource constraints and the risks associated with patients accessing healthcare. In Australia the Urological Society of Australia and New Zealand (USANZ) has also published guidance [15], though it is somewhat less proscriptive than the international equivalents.

Being a purely observational study based on registry data this study has a number of limitations. Most significantly owing to the nature of the data being used it is entirely reliant on the quality of the data being submitted to the DHS, and is reliant on both clinicians and administrative staff to accurately record the MBS Item Number for the test being performed, however unless there is a reason to believe that the quality of this data entry is significantly different in 2020 as compared to preceding years there is no reason to believe that this will have imparted a systematic bias upon the conclusion.

Similarly, with regards to the modelling performed in order to predict the testing rate in 2020 we were limited to publicly available open-access information, and the only reliably available predictive variable to use in regression analysis was population. As such the R^2^ values in our models were relatively low varying between 0.29 (for biopsies performed at a national level) through to 0.6 (for biopsies performed in Victoria) indicating a relatively high degree of heterogeneity. As can be appreciated in Figs. 1 and 2 there is heterogeneity in the testing rates, and although not shown the rate of population growth was relatively consistent throughout this period, indicating that other factors are affecting the rate of testing. As such we feel that caution should be taken in considering the significance of the difference between the predicted and actual testing rate, and that our modelling should be treated as more descriptive rather than inferential. However, as we can observe great similarities between the predicted test usage for 2020 and the average test usage in the period 2015–2019 we suspect that the results can be treated as compelling.

## Conclusion

The current pandemic has had a widespread reach across Australia, with varying impact across each state and territory. Contrary to the trends across the world, our data suggest that during 2020 in Australia most areas remained unaffected in terms of prostate cancer testing. The exception to this was Victoria, which had statistically significant decrease in the number of PSA tests performed, corresponding with the periods of lockdown that occurred in the state, however even then the absolute decrease was relatively limited.

In these unprecedented times, we believe that it is reassuring for urologist to see that prostate cancer screening testing remains largely unchanged, and thus may have less implications for future cancer care then anticipated. However, as the trajectory for COVID-19 continues to evolve, screening protocols and recommendations may be subject to change and thus need frequent re-evaluation.

## Data Availability

Data was extracted from the Department of Human Services (DHS) website (http://medicarestatistics.humanservices.gov.au/statistics/mbs_item.jsp) for Medicare Benefits Schedule (MBS) item numbers for tests pertinent to detection of Prostate Cancer. This data is deidentified and publicly available.
